# The impact of repeated mass antigen testing for COVID-19 on the prevalence of the disease

**DOI:** 10.1007/s00148-021-00856-z

**Published:** 2021-06-29

**Authors:** Martin Kahanec, Lukáš Lafférs, Bernhard Schmidpeter

**Affiliations:** 1 CELSI, UEBA and GLO, Central European University, Quellenstrasse 51, A-1100 Vienna, Austria; 2 Central European Labour Studies Institute (CELSI), Zvolenská 29, 821 09 Bratislava, Slovakia; 3 University of Economics in Bratislava, Dolnozemská cesta 1, 852 35 Bratislava, Slovakia; 4 Global Labor Organization, Leimkugelstr. 6, 45141 Essen, Germany; 5 Department of Mathematics, Faculty of Natural Sciences, Matej Bel University, Tajovského 40, 97401 Banská Bystrica, Slovakia; 6 Department of Economics, Johannes Kepler University Linz, Altenbergerstr. 69, 4040 Linz, Austria; 7 RWI, Leibniz Institute for Economic Research, Essen, Germany; 8 IZA, Institute for the Study of Labor, Bonn, Germany

**Keywords:** COVID-19, COVID-19 policies, Antigen testing, Mass testing, Non-pharmaceutical interventions, D04, I18, J22

## Abstract

In the absence of effective vaccination, mass testing and quarantining of positive cases and their contacts could help to mitigate pandemics and allow economies to stay open. We investigate the effects of repeated mass testing on the COVID-19 pandemic caused by the SARS-CoV-2 virus, using data from the first ever nationwide rapid antigen testing implemented in Slovakia in autumn 2020. After the first round of testing, only districts above an ex ante unknown threshold of test positivity were re-tested. Comparing districts above and below the threshold, we provide evidence that repeated mass antigen testing can temporarily reduce the number of new infections. Our results suggest that mass testing coupled with the quarantining of positive cases and their contacts could be an effective tool in mitigating pandemics. For lasting effects, re-testing at regular intervals would likely be necessary.

## Introduction

More than one year after the first documented cases of COVID-19 in Wuhan, China, in 2019, most countries are still struggling to contain this highly contagious and severe disease caused by the SARS-CoV-2 virus (see Qiu et al. 2020, for an early study on the transmission of COVID-19). According to data provided by Johns Hopkins University, more than 120 million people around the world have been infected and almost 3 million people have died of COVID-19 as of March 30, 2021.^1^ To protect their most vulnerable citizens and to slow the spread of the disease, many governments have imposed strict policy measures, such as social distancing requirements, stay-at-home orders, and local and nationwide lock-downs. While there is evidence that some of these policies have been successful in at least slowing the number of infections (e.g., Chernozhukov et al. 2020; Bonacini et al. 2021), they also have both directly and indirectly affected labor supply and demand, investment, consumption, and other economic variables, taking a heavy toll on economies. The world GDP is projected to have fallen by more than 4% in 2020 (IMF 2020). The projected decline in GDP is even more pronounced for advanced economies. Economic distress caused by the pandemic policy measures has also affected broader aspects of peoples’ lives and well-being (e.g., Arenas-Arroyo et al. 2021; Brodeur et al.^2021^).

Facing such detrimental effects on both theeconomy and society, and with the prospect of widely accessible vaccination still distant — especially for low-income countries — policy makers have been looking for alternative ways of containing the pandemic. Mass testing for COVID-19 has received particular attention as a potential tool for suppressing the pandemic.^2^ Regional and local mass antigen testing has been carried out in several countries, such as the UK, China, South Korea, Austria, Luxembourg, and Slovakia. Evidence on whether and how (repeated) mass testing can work to mitigate pandemics is scant, however. Informing policy makers on the question of whether mass testing can be an effective policy tool to re-open the economy during a pandemic is hence an urgent call.

Proponents of mass testing maintain that it is a cost-efficient policy for identifying and quarantining potentially infectious individuals. This direct effect of testing would in turn help reduce the number of cases and the spread of the disease (e.g., Pavelka et al. 2021). If mass testing could accomplish this, costly social-distancing policies could be eased or lifted, schools and the economy could remain open, and far-reaching social and psychological costs could be averted.

In contrast, opponents of mass testing argue that it may create a false sense of security and may lead individuals to behave less carefully, as argued by, for example, Mahase (2020). Cheaper rapid antigen (Ag) tests, which are often used in mass testing events, generally have lower sensitivity and specificity compared with the more expensive reverse transcription polymerase chain reaction (PCR) tests, likely leading to higher rates of false negatives and false positives in mass antigen testing. This could undercut the credibility of such testing and anti-COVID-19 measures in general. In contrast to the original intention of mass testing, a larger share of false positive tests would incorrectly confine a correspondingly larger share of workers in quarantine, putting an unjustified pressure on the economy (Pettengill and McAdam 2020). Potentially even more detrimentally, negative test results, whether true or, even worse, false, would likely reduce people’s caution in social contacts and increase risky behavior, leading to an increased spread of the disease. As the relative shares of false and true positives and negatives depend on disease prevalence, so too do the relative strengths of these direct and indirect effects (Dinnes et al. 2021). Specifically, the benefits of testing tend to be smaller and the signals more noisy in low-prevalence, asymptomatic populations, and vice versa (Dinnes et al. 2021). In light of these arguments, the overall effect of mass testing on disease prevalence is an empirical question. From the perspective of effective suppression of pandemics, it is important to know whether repeated mass testing can in practice suppress the pandemic and if so, for how long the potential benefits may last.

In this study, we evaluate the impact of repeated mass antigen testing coupled with quarantine measures on the spread of the COVID-19 pandemic using epidemiological data from Slovakia. Over two days in autumn 2020, Slovakia was one of the first countries in the world to conduct a countrywide mass testing event using rapid antigen tests. All those who received a positive test result, their household members and self-traced recent contacts (from the past two days), as well as those without a valid negative test, were required to quarantine for ten days. One day after the mass testing event, the government announced that in districts where the share of positive COVID-19 tests was equal to or above 0.7% a second round of mass testing would be conducted. As we argue below, a lot of uncertainty surrounded whether the second round would take place, and if so, what criteria would be used to select districts for testing or whether it would be conducted nationwide. Given these uncertainties and the minimal, one-day difference between the time when the results from the first round became available and the announcement of the selection threshold, we argue that the chosen threshold was ad hoc and ex ante publicly unknown, and as a result, as good as random for the purpose of this study.

In our empirical approach, we exploit this unique mass-testing setting in Slovakia to evaluate the potential benefits of repeated mass testing. We compare districts above and below the announced threshold using a difference-in-differences framework.^3^ Our empirical approach requires that districts above and below the threshold would have followed a similar infection trajectory absent repeated mass testing. In light of the discussion about rapid antigen tests above, to be able to identify the impact of repeated mass antigen testing on the spread of the disease, we also need to rule out any dynamic unobservable effects within districts, such as systematically changing differences between districts above and below the threshold in the sensitivity rates of the rapid antigen tests or in the composition of populations participating in testing.^4^

We find that in those districts above the threshold, the measured number of infections fell on average by up to about 30% and the reproduction number, which measures the number of secondary infections per case generated in the population, decreased by about 0.3 two weeks after the second mass testing event, compared to districts below the threshold. Exploring the dynamics behind these effects, our results indicate a maximum reduction in COVID-19 incidence around 15 days after the second mass testing and a reversal to zero afterward. Three weeks after the second round of mass testing all the measured effects disappeared.

To investigate the robustness of our results, we conduct a wide range of robustness tests. We show that our results are qualitatively similar when discarding districts further away from the threshold, which arguably might have fundamentally different infection dynamics. In addition, we control for several potentially confounding factors, show that our results are not driven by relatively large districts in terms of population size, and provide support for the assumption that the re-testing threshold was practically as good as random.

We make several contributions to the small but rapidly growing literature on the effects of mass antigen testing on the COVID-19 pandemic, which we review in Section 2.^5^ To the best of our knowledge, this is the first study systematically evaluating the potential benefits of repeated mass antigen testing, exploiting the unique setting surrounding the mass testing events in Slovakia using a difference-in-differences identification strategy.^6^ Our strategy enhances the external validity of the estimation results while relying on arguably weaker assumptions than model-based evaluation methods (e.g., Atkeson et al. 2020). Our approach also allows the investigation of possibly dynamic effects of repeated mass testing. How long potential benefits of mass testing last is an important parameter in guiding decision makers. This parameter has so far only received limited attention in the empirical literature.

The results of this study are of interest for policy makers in light of the question of how mass testing together with strict contact tracing and quarantine measures can help mitigate pandemics. First, we show that repeated mass testing can be an effective policy tool for decreasing the spread of the disease when coupled with an effective quarantine regime of positive cases and their contacts. Second, our results indicate that the mitigating effect is temporary. While we are not able to disentangle to what extent such a dissipation of the effects is driven by people’s behavioral adjustment to testing or test results, the epidemiological properties of the disease, or other channels, this result implies that mass testing would need to be conducted on a regular basis if a sustained mitigation of the pandemic were to be achieved.

The paper proceeds by discussing the literature on mass testing and our contribution to it in the next section. We then describe the institutional setting and the mass antigen testing events that took place in Slovakia in the autumn of 2020 in Section 3. The empirical approach as well as the data used are outlined in Section 4. In Section 5, we report and discuss our main estimation results. Section 6 presents various robustness and falsification tests. Section 7 discusses the contribution of this study, its limitations, and concludes.

## Literature review

It has been suggested that rapid antigen testing may play an important role in mitigating the pandemic (Baqaee et al. 2020). The low price and broad accessibility of these tests and the relatively short time needed until test results are available make them a potentially useful and likely cost-effective tool (Atkeson et al. 2020). In addition, recent evidence points to a relatively high sensitivity and specificity of the best antigen tests available on the market, even if the rates of false positivity and false negativity are not trivial (see, e.g., Mina et al. (2020b)). Despite these recent developments, the potential benefits of rapid mass testing as a policy instrument for mitigation of the COVID-19 pandemic have until recently received only limited attention (Mina et al. 2020a).

Several studies develop theoretical models to evaluate the possible effects of antigen testing. Using a behavioral Suspected-Infected-Recovered (SIR) model for the USA, (Atkeson et al. 2020) propose that a simple and low-cost two-step procedure may yield the best results from a cost-benefit perspective; for example, a low specificity antigen testing followed by a high-specificity confirmatory antigen testing of those who tested positive. The authors underscore that cost-effectiveness of mass testing critically depends on public compliance with quarantine of those who test positive (and their contacts) and on whether mass testing increases or decreases risky behaviors.

Mina et al. (2020a) develop a theoretical model to study the effect of testing on infections, explicitly modeling the effect of social distancing and social activity as network formation problems. They argue that testing and isolating can work but also that testing increases the range of social networks as individuals feel more secure. Using a theoretical model on reopening universities, Platiel et al. (2020) argue that rapid testing can be effective, but that testing has to be conducted in very short-time intervals. Their results indicate that students need to be screened every 2 days, in addition to general vigilance and good prevention practice. Their conclusions are derived for a hypothetical cohort of students, however.

Pettengill and McAdam (2020), in contrast, doubt whether rapid antigen testing can mitigate the COVID-19 pandemic. First, they argue, antigen testing produces nontrivial numbers of false positives, which can undercut the credibility of testing programs and compliance with quarantine orders. This is especially the case if false positivity is revealed to the tested by, for example, confirmatory PCR testing. Second, using cheaper and faster, but less precise tests may also put a large drag on the economy by placing a lot of workers wrongly in isolation. Third, the imperfect sensitivity of antigen testing implies that a significant numbers of infected individuals do not get identified. This may increase their risky behavior and worsen the pandemic. In light of these theoretical arguments, the effect of mass testing on mitigating pandemics is ambiguous.

Empirical studies about the impacts of (antigen) testing on the spread of COVID-19 are scant. Callaway and Li (2020) evaluate Tennessee’s open testing policy using a bounding approach that allows for non-randomly missing test data. Using bordering states as controls, they show that increased accessibility of testing reduced overall cases (which are not fully observed), confirmed cases, and work trips among counties with fast-growing numbers of confirmed cases.

A few studies look at the mass antigen testing campaign in Slovakia. Holt (2021) summarizes Slovakia’s experience with the autumn mass antigen testing, highlighting the issue of the relatively low sensitivity and specificity of antigen tests, potentially resulting in a high incidence of false positive and false negative results. As no systematic retesting with PCR tests was conducted, little is known about the true significance of this problem. Bod’ová and Kollár (2020) study the spatial patterns of the COVID-19 epidemic in Slovakia. They conclude that the mitigating effect of repeated antigen testing increased with the measured prevalence of the disease in the first round of testing.

Closely related to our study is the work by Pavelka et al. (2021), who explore the impact of mass antigen testing in Slovakia by comparing the spread of the disease across districts and in different rounds of the mass testing events. Complementing a statistical model with a microsimulation approach, the authors find that the decrease in prevalence compared to a scenario of unmitigated growth cannot be fully explained by non-pharmaceutical interventions implemented before the mass antigen testing. They interpret this difference as an impact of antigen testing and the ensuing quarantine of positively tested individuals on the spread of the disease. Mahase (2020) reviews the study, pointing out that it does not disentangle the effects of testing from those resulting from the lockdown measures and suggests that its external validity may be limited.

Our approach differs from Pavelka et al. (2021) in several ways. First, we use different outcome measures. While Pavelka et al. (2021) use the results from the mass antigen testing, we measure the spread of the disease using data from standard passive surveillance PCR and antigen testing that was conducted independently of the mass testing. As the positive cases from the first round of the mass testing and their close contacts were quarantined for ten days and were not included in the second round of mass testing, the sample of individuals tested in the first and second round differed. This complicates identifying the impact of repeated mass testing by comparing the results from different rounds of testing, as the difference in test positivity between the second and the first round is partly driven by the changing sample of individuals. In addition, by using daily data from standard testing, our approach also permits us to study the evolution of the effects of mass testing over time.

Mass testing could have distorted our measures of the pandemic based on daily results from passive surveillance testing in the proximity of the mass testing events, however. Specific types of people might have been selecting into (or out of) the mass testing rather than the standard surveillance testing. However, such substitution is rather implausible beyond a few days and hence we argue that our approach considering the evolution of the effects of mass testing over the three weeks following the second and final round of mass testing is sufficiently salient in this regard. In addition, given the nature of our research design, such distortions would affect our results only if they had systematically heterogeneous effects on different districts at the time of measurement.

Second, while Pavelka et al. (2021) estimate the effect of the first round of mass testing on the sample of repeatedly tested districts and obtain the effect of the pilot testing for the four pilot districts, we focus on the effects of the second round of testing and exclude the pilot districts. This enables us to extricate the effects on the pandemic of the second round of the mass testing campaign and the related measures implemented in the re-tested districts from the effects of any measures implemented nationwide, including the lockdown, school closures, or bans on gatherings and commercial and sport activities, or any other nationwide trends.

## The COVID-19 situation and mass testing in Slovakia

### The run-up to the mass testing

Slovakia’s experience with the COVID-19 pandemic can be characterized by two rather different phases, roughly divided by the end of August 2020. The first COVID-19 case in the country was recorded on March 6 and the first death on March 30, 2020. By August 31, 2020, the country recorded 3989 total cases from passive surveillance PCR testing and 33 deaths (IHA 2021). The country’s relative success could probably be credited to its early non-pharmaceutical interventions: schools and universities in Bratislava, the country’s capital, were closed within less than a week after the first case, border controls and mandatory quarantine for people returning from abroad were introduced, and non-essential shops were closed. Within ten days from the first case, schools in the entire country were closed, face-masks became mandatory in public spaces, and international public transport was suspended. The shock from the pandemic and the example of public figures wearing face-masks likely contributed to a high level of compliance with social distancing measures. The social distancing measures were gradually lifted or eased during the summer.

The end of summer 2020 marked a turning point. By the end of September, the number of cases had increased to 10,938 and by October 23, 2021, one week before the first round of mass testing, 40,801 cumulative PCR-positive cases had been identified. In an effort to bring the pandemic under control, Slovakia implemented several containment measures, such as partial school closings and restrictions on indoor hospitality as well as on leisure activities. Unlike in other countries where social distancing measures were often imposed at a local level, in Slovakia, non-pharmaceutical interventions were implemented nationwide with no regional variation. To ensure compliance with the measures, police conducted random checks. In spite of the gradually tightened social distancing measures, on October 29, 2020, the increase in the number of cases reached 3363 on a single day.

As the measures already in place were considered not to be sufficient to curb the spread of the pandemic, in October and November 2020 Slovakia became the first country in the world to announce and implement nationwide mass rapid antigen testing intended to detect and quarantine COVID-19 cases early and curb the spread of the disease. With a total population of 5.45 million people, residents aged between 10 and 65 years as well as older adults in employment, or about 80% of the total population, were eligible for voluntary rapid antigen mass testing. In total, 5,276,832 SD Biosensor Standard Q rapid antigen tests were administered (Pavelka et al. 2021).

During the week prior to the first mass testing event, the government implemented pilot testing from October 23 to 25, 2020, in four districts (Bardejov, Dolný Kubín, Námestovo, and Tvrdošín) and asked citizens in all of Slovakia to limit their movement. The four districts were chosen on the basis of their particularly adverse epidemic situation at the time. The first round of nationwide testing took place on October 31 and November 1, 2020 (Round 1). Around twenty thousand healthcare professionals and forty thousand army personnel and volunteers helped to test residents of all the country’s 79 districts. Citizens with positive test results, members of their households, and their self-traced recent contacts (over the past 2 days) had to quarantine for ten days. Even though participation in mass testing was voluntary, those who did not participate were also obliged to quarantine for ten days. Employees had to present a negative test certificate to their employers to be able to be physically present at work. To enforce the quarantine, random inspections in public places were conducted and individuals who were unable to present a certified negative test result were fined up to 1659 Euro, corresponding to about 1.5 times the average national monthly wage.^7^

Shortly after the first testing round, on November 2, 2020, the government announced that in all districts with the rate of test positivity in Round 1 of 0.7% or higher a second round of mass testing was to be conducted on November 7 and 8, 2020 (Round 2). Applying this ex ante unknown threshold, the second round of testing was conducted in 45 districts. As it was the case during the first round, participation was voluntary but the same restrictions were imposed on non-participating citizens and those who were tested positive. Given the strict enforcement policy, the participation rate of the eligible population in the pilot and the two waves of testing ranged between 84 and 87% (Pavelka et al. 2021). As a result of the mass testing efforts, 50,466 individuals with positive test results were identified, with the rate of test positivity varying from 3.91% during the pilot to 1.01% in the first round of mass testing and 0.62% in the second round. (Pavelka et al. 2021)

It is important to note that the decision about the second round of mass testing and the specific test-positivity threshold based on which districts were selected into retesting were announced only a few days prior to Round 2. As late as on October 30 and 31, the minister of defense and minister of the interior expressed doubts about whether the second round would take place.^8^ On October 31 and November 1 the prime minister stated that the second round would take place, expressing his preference for nationwide retesting but admitting that “one, two, or three” districts with “extremely good results” could be exempted.^9^ On November 2, the government approved the second round and the threshold of 0.7%, even though a council of experts suggested a different threshold of 1.5%.^10^ Whether a given district was included in the second round of testing was unknown to most district citizens and authorities until just a few days prior to the second round of testing. The official list of districts required to participate was in fact announced only on November 3, 2020, and the results from Round 1 were not published before that date. This enables us to treat the second round as a quasi experiment, with some districts “treated” and others “non-treated”.

Following the design of the different testing regimes, we consider all districts with two mass testing events to be in our treatment group. Districts that only participated in one mass testing event constitute our control group. Due to their unique setting and their particular epidemiological situation, we do not consider the four districts participating in the pilot scheme in our analysis.^11^

**Fig. 1:**
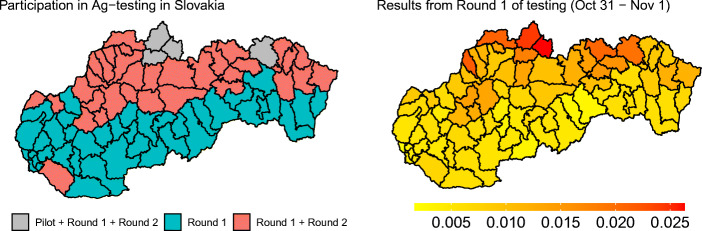
Participation of different districts in mass Ag testing and results from the first round

In Fig. 1, we provide an overview over the location of the different districts in our sample and the share of the persons who tested positive in the first round. As the left hand side of the figure shows, most of the districts subjected to two rounds of testing are located in the north of Slovakia. While this could suggest a North-South geographic pattern of the spread of the disease in Slovakia, a much more nuanced pattern emerges when looking at the share of positive tests during the first round by district, presented on the right hand side. There is substantial variation both within and between districts with one mass testing event and districts with two mass testing events. In fact, the difference in first-round test positivity between two districts where one district is selected for the second round of testing while the other one is not can be as little as 0.02 percentage points, as in districts Nitra and Zvolen, for instance. These observations support our assumption that the applied threshold of 0.7% was arbitrarily chosen.^12^ We provide additional information about the epidemiological situation before the mass testing in Appendix 1.

### The situation after the mass testing

Looking at the evolution of the spread of COVID-19 after the mass testing, the 7-day moving average of new infections decreased for about 3–4 weeks following the mass testing. At the end of November, however, a new, more pronounced wave of infections started, peaking at the end of 2020 and beginning of 2021. Whereas mid-January witnessed a decline in the numbers of new infections, yet another wave, somewhat less pronounced than the two previous ones, started at the end of January 2021, peaking in early March 2021 and slowly withering away since then (based on data through April 10, 2021). These waves of COVID-19 infections took a heavy toll on people’s lives, as the number of recorded COVID-19 deaths surpassed 10,000 (1900 per million citizens) in early April 2021 (IHA 2021).

It would be misleading to interpret the decline in the number of daily cases reported during the three weeks following the autumn mass testing solely as direct evidence of the mass-testing campaign’s impacts. Indeed, several potentially confounding nationwide interventions were implemented during the weeks preceding the reversal of the epidemiological trends at about the time of the mass-testing campaign: gatherings of more than 50 people and wedding receptions were banned on October 1; secondary schools were closed on October 12; gatherings of more than 6 people and indoor leisure activities were banned and indoor hospitality (including restaurants, cafes, patisseries, pubs, and bars) was closed on October 15; a lockdown banning non-essential movement and outdoor activities was implemented on October 24; and a partial closure of primary schools (grades 5 and higher) took place on October 26. The lockdown was lifted on November 15 and several additional activities (visiting some sport facilities, theaters, churches, on-site instruction for pupils from disadvantaged backgrounds without access to distant instruction) were allowed as of November 16, 2020. It is precisely the disentanglement of the effects of Round 2 of mass testing (quarantining those who tested positively, their household members and self-traced contacts, as well as those who were not tested for ten days, and distributing negative test certificates to all those who tested negatively) from the effects of these nationwide measures and other nationwide trends on the epidemiological situation in the treated districts that this paper attempts to do.

Similarly, the adverse evolution of the pandemic during winter 20/21 could be seen eventually as an indication that the autumn mass testing campaign did not help and perhaps even worsened the epidemiological situation in Slovakia. While such interpretation could be broadly consistent with the observed epidemiological trends around the turn of the year, many other factors or interventions could have driven the observed patterns. The effects of varied behavioral responses of people to the epidemiological situation in the district, mobility across districts, the winter season with more people staying indoors more often and for a longer time period, uneven introduction of new variants of the COVID-19 virus across districts, super-spreading events resulting in explosive growth in some districts, variation in responses of local authorities to the pandemic situation, reversion to mean over a long time horizon, and a range of other factors likely introduce significant noise in the data. We looked at longer-term trends in our data, but we judged that a growing level of noise in the data due to outbreaks of the pandemic in several districts and the possible accumulation of such confounding factors precluded meaningful analysis of the long-term effects of the mass testing campaign.

## Data and empirical approach

### Data

We make use of data provided by the Institute of Health Analyses (IHA), an analytical unit of the Ministry of Health of the Slovak Republic. For all 79 districts in Slovakia, the IHA collects data on the daily number of infections within a district. In addition, it also collects information on the total number of conducted and positive PCR and rapid antigen tests for 72 districts.^13^ After removing the four districts that were included in the pilot testing, we are left with 68 districts in our analysis. For all these 68 districts, we obtain the daily number of positive tests.^14^

From the data, we construct two measures that reflect the spread of COVID-19. Our first measure is the 7-day rolling average of infections on the district level. The 7-day rolling average is less noisy and more robust to intra-week variation in testing intensity compared to other measures, such as daily cases.

Our second measure is the reproduction number *R*_0_. This measure reflects how many additional people one person with COVID-19 is expected to infect directly. *R*_0_ can therefore be thought of as capturing how contagious or transmissible COVID-19 is. As in epidemic nowcasting in Germany (Hamouda et al. 2020), we calculate *R*_0_ at time *T* as $\left ({\sum }_{\tau =T-7}^{T-1} y_{\tau } \right ) / \left ({\sum }_{\tau =T-12}^{T-6} y_{\tau } \right )$, where *τ* is a day and *y* the numbers of new infections. This formula for *R*_0_ uses information on infections up to 12 days prior to time *T*. It is therefore a more backward looking measure than the other measure that we use, the 7-day rolling average number of daily cases.^15^

We also collected district-level characteristics such as the overall participation rate in the first mass testing event (Round 1), the overall population, population density, and the economic conditions prior to the first mass testing event as proxied by the local unemployment rate (data provided by the Slovak Statistical Office). Table 1 provides summary statistics for our sample and the difference in background characteristics between treatment and control groups. From the summary statistics, one can see that the participation rate in the first round was nearly identical in the treated and control districts. The participation rate was also quite high, with around 60% of the overall population on average.^16^ We also do not find large differences between treated and control districts when looking at local economic conditions. Districts in the control group are slightly more densely populated compared to districts in our treatment group, however. In our robustness checks, we investigate the sensitivity of our results to population density. Overall, the similarity between the treated and control districts is reassuring for our empirical design.

**Table 1:** Summary statistics

Group	Pop	Type	Dist. Pop.	Pop. density	Unempl.	Ag-R1	Ag-R2	Cases	R0	R1 part.
Treated	2.72mil	Mean	66416	104.4	5.63%	1.49%	0.64%	5.55	1.28	59.9%
(N = 41)		S.D.	(38952)	(44.1)	(3.09%)	(0.57%)	(0.28%)	(2.36)	(0.42)	(5.9%)
Non-treated	2.51mil	Mean	93309	188.4	5.53%	0.5%		2.67	1.47	61.8%
(N = 27)		S.D.	(85408)	(311.4)	(3.82%)	(0.12%)		(1.38)	(0.78)	(9.6%)
Pilot	216k	Mean	54101	82.5	5.64%	1.72%	0.69%	4.07	0.45	57.2%
(N = 4)		S.D.	(19787)	(6.7)	(2.99%)	(0.24%)	(0.15%)	(0.93)	(0.17)	(4.4%)

*Ag-R1*/*Ag-R2* stand for percentage of positive Ag cases from Round 1 and Round 2 of mass-testing, respectively. *Cases* are 7-day averages of total (PCR and Ag) infections at the time of Round 1. *R0* is a simplified *R*_0_ for total. (PCR and Ag) infections. *R1 part* is participation in the first round — percentage of total population

### Empirical approach

To estimate the impact of repeated mass testing on the spread of COVID-19, we consider a difference-in-differences model. In our main analysis, we consider two time periods: the pre-period for which *t* = 0 (Nov 8, 2020) and the post-period for which *t* = 1 (Nov 22, 2020).

1 $$ y_{it} = \beta_{0} + \beta_{1} (testedR2_{i} \cdot t) + \beta_{2} t + \beta_{3} testedR2_{i} + \epsilon_{it}, $$

where *y*_*it*_ is the outcome, either the 7-day rolling average of new infections or *R*_0_, measured in district *i* at time *t*.^17^ The variable *testedR*2 is an indicator variable taking the value of 1 if district *i* participated in the second round of mass testing (was treated) and 0 otherwise (was not treated, control).^18^

In Eq. (1), *β*_1_ is our parameter of interest. It measures the impact of repeated mass testing on our outcome variables under two assumptions.^19^ First, as mentioned above, our identification strategy is based on the parallel trends assumption that absent of the second round of mass testing the outcome would have evolved similarly in the treatment and control groups. In order to test the robustness of our results with respect to this assumption, we estimate our empirical model on a restricted sample of only those districts that were relatively close to the treatment threshold and where the epidemiological situation was more similar. Furthermore, as shown in Table 1, the average treated and non-treated districts (above and below the retesting threshold) were relatively similar.

Another underlying assumption of our empirical model is that there have not been any systematically different changes over time in within-district characteristics in those districts that were retested and those that were not. For example, we need to rule out that the composition of individuals taking the test systematically and differently varied within treated and non-treated districts over time. Similarly, we also need to rule out systematic changes over time in (average) compliance with policy measures imposed or the sensitivity of the rapid antigen tests used.^20^

Finally, we assume that it takes time until the effects of the second round of testing can materialize. Specifically, our baseline approach is based on the premise that it takes several days until an individual develops any symptoms, they register and obtain a date for testing, and the results are reported in the official statistics. In line with the literature reviewed in Section 2, we assume that this process takes between 8 and 14 days. In our analysis, we analyze the sensitivity of our results with respect to the duration of this lag by reporting the estimated effects for the whole range of possible lags up to three weeks after the second round of testing. This approach also enables us to shed light on the timing patterns of the effects of mass antigen testing.

## Results

### Descriptive evidence

**Fig. 2:**
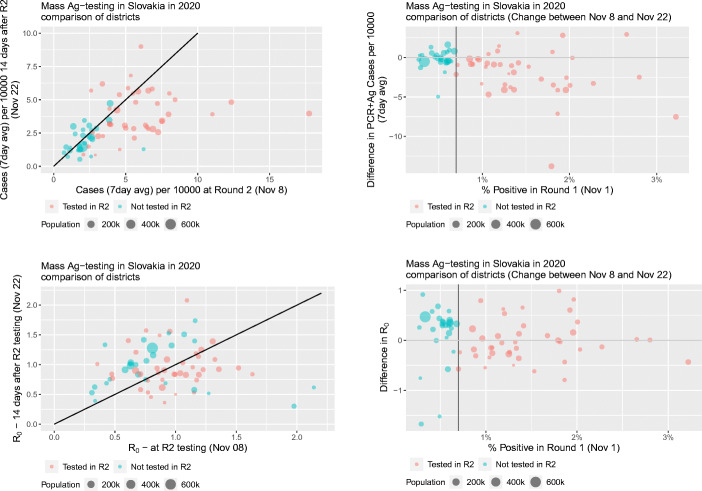
Association between the results from the first round of mass testing and the 7-day average and *R*_0_ two weeks after Round 2

We begin by providing descriptive evidence about the prevalence of COVID-19 in Slovakia between the first and second round of mass testing. Both measures of the pandemic that we use, the 7-day average and *R*_0_, are calculated per 10,000 inhabitants based on PCR and antigen tests from passive surveillance testing. In the top left panel of Fig. 2, we plot the relation between the 7-day average of (normalized) positive cases 14 days after the first mass testing, on November 22, against the 7-day average of positive cases at the start of the second round of mass testing on November 8. The red circles represent districts in our treatment group (retested) and the green circles represent districts in the control group (no Round 2). The size of the circles depend on the population of the districts, with larger circles representing larger districts. The bottom left panel follows a similar logic for *R*_0_.

Using the same measures, in the right panels of Fig. 2, we plot the changes in the 7-day average of positive cases (top panel) and changes in *R*_0_ (bottom panel) between November 8 and November 22 against the share of positive tests after the first mass testing event. As above, red circles represents treated districts and green circles depict control districts.

Looking at the average number of cases in the top-left panel, we observe that control districts are lined up along the 45-degree line whereas most districts which were re-tested lie below the 45-degree line. In other words, re-tested districts experienced in general a larger drop in infections than those exempted from the second round. This effect can also be seen when looking at the changes in the average number of infections in the top-right panel. In general, treated districts have seen a larger drop in infections while changes in the number of positive cases in our control group are centered around zero difference.

Interestingly, when looking at *R*_0_ in the bottom panels, we see that control districts actually experienced an increase in *R*_0_ between November 8 and November 22, as opposed to no systematic change in the treated districts. As *R*_0_ is backward looking, this increase points toward the likely short-term effects of mass testing.^21^

**Fig. 3:**
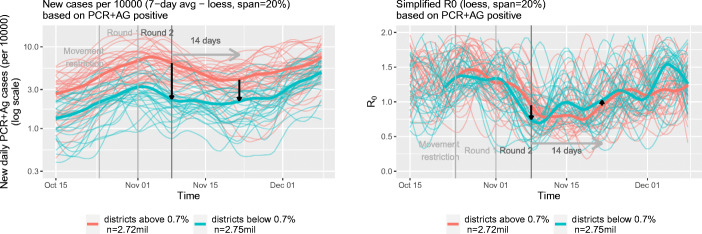
Evolution of infections and *R*_0_ in the districts below and above the threshold 0.7% for the full sample

In Fig. 3, we report trends in the 7-day average of positive cases and the reproduction number *R*_0_ in treated (red) and non-treated (green) districts. As above, *PCR+AG positive* stands for infections detected by PCR and antigen tests. The thick red line represents averages for the treated districts and the thick green line averages for the control districts. Consistent with the patterns observed in the left panels of Fig. 2, we observe that differences in the average number of cases decreased between the first and second mass testing event. The graphs also support our parallel trends assumption. Prior to the first mass testing event — the timing of our treatment — the average number of infections developed in a very similar pattern in our treatment and control districts.

In the right panel of Fig. 3, we depict the development of *R*_0_ in the treatment and control groups over time. One can see a clear increase in this measure for control districts after the second round of testing. As discussed above, this increase points toward the relative short-term benefits of mass testing.

While these simple comparisons provide some initial insights into the possible epidemiological benefits of mass testing, there are also obvious shortcomings. For example, the districts in the treatment and control group might differ in unobserved characteristics, such as compliance with mobility restrictions and other non-pharmaceutical interventions. In the next section, we evaluate the potential benefits of repeated mass testing in a more formal way.

### Measuring the impact of retesting

In this section, we present estimates from the difference-in-differences model defined in Eq. (1). We estimate this model using the two dependent variables introduced above, the 7-day average number of positive PCR and antigen tests per 10, 000 citizens and *R*_0_, as well as the logarithms of these variables. Notice that because of its backward-looking nature, we would expect our results for *R*_0_ to be noisier than results for the average number of cases. We think that using *R*_0_ as an outcome is nevertheless interesting given its popularity in the media and the literature.

Table 2 presents the results. Regressions are weighted by district population size.^22^ Looking at column 1, we see that the second wave of mass antigen testing was associated with a reduction of the 7-day average in infections measured 14 days after Round 2 by approximately 2.3 daily cases per 100.000 inhabitants. This constitutes quite a sizable reduction of 36%, as can be seen from our estimates reported in column 2 for the logarithmic transformation.

**Table 2:** Effect of repeated mass testing on COVID-19 infections — full sample

	Dependent variable			
	Cases	log Cases	*R*_0_	log *R*_0_
	(1)	(2)	(3)	(4)
Tested in R2 × Post period	− 2.252^∗∗∗^	− 0.358^∗∗^	− 0.279^∗∗∗^	− 0.314^∗∗∗^
	(0.632)	(0.144)	(0.100)	(0.112)
Tested in R2	4.047^∗∗∗^	1.005^∗∗∗^	0.186^∗∗∗^	0.235^∗∗∗^
	(0.447)	(0.102)	(0.071)	(0.079)
Post period	− 0.125	− 0.092	0.271^∗∗∗^	0.306^∗∗∗^
	(0.446)	(0.101)	(0.071)	(0.079)
Intercept	2.243^∗∗∗^	0.739^∗∗∗^	0.787^∗∗∗^	− 0.302^∗∗∗^
	(0.315)	(0.072)	(0.050)	(0.056)
Observations	136	136	136	136
R^2^	0.463	0.535	0.105	0.114

Districts weighted by their population size

^∗∗∗^*p* < 0.01; ^∗∗^*p* < 0.05; ^∗^*p* < 0.1

In columns 3 and 4 of Table 2 we also report the impact of repeated mass testing on the reproduction number *R*_0_ and log *R*_0_ respectively. Our estimates suggest that the second round of testing decreased simplified *R*_0_ by approximately 0.28 more in the treated districts than the non-treated ones, corresponding to a reduction by 31%. Loosely speaking, these results imply that repeated mass testing reduces the number of people to whom ten infected persons pass on the infection two weeks after the second round by 2.8. However, given the backward looking nature of *R*_0_, we caution to interpret these results with due care.

While the setting in Slovakia was unique, it is nevertheless worth comparing our results to the impact estimates for non-pharmaceutical interventions in the literature. Using data for the USA, Chernozhukov et al. (2020) estimate that face masks reduced the weekly growth rate in the number of cases by around 10%. They also find that stay-at-home-orders reduced the number of new cases by 6 to 63%. Putting our findings into perspective, they would imply that repeated mass testing together with quarantining of those that test positive, their household members and recent contacts, as well as those that do not participate in the testing has the potential to be more effective than mandatory mask wearing and may even be as effective as some stay-at-home orders, at least in the short run.

Our estimates differ from those estimated by Pavelka et al. (2021), however, who estimate a decrease in the prevalence within one week after mass testing of up to 70%. As we describe in Section 2, the differences could be attributed to differences in the outcome measures used and the treatment of interest, as our estimates reflect the impact of the second round of mass testing only. Therefore, we see our estimates as complementary to the results of Pavelka et al. (2021).

### Distance to threshold and regression-to-the-mean

One concern about our results reported in the previous section could be that districts further away from the 0.7% threshold might be fundamentally different compared to those closer to it. Districts with a very high share of positive cases in the first round of mass testing might exhibit different infection dynamics compared to those districts with a very low share of positive tests. This might lead to a nontrivial bias in our estimates. In this section, we assess the robustness of our results considering only districts (relatively) close to the 0.7% threshold. This comes at a cost, however; given our already relatively small sample, one would expect less precise estimates when excluding districts from our estimation sample.

**Fig. 4:**
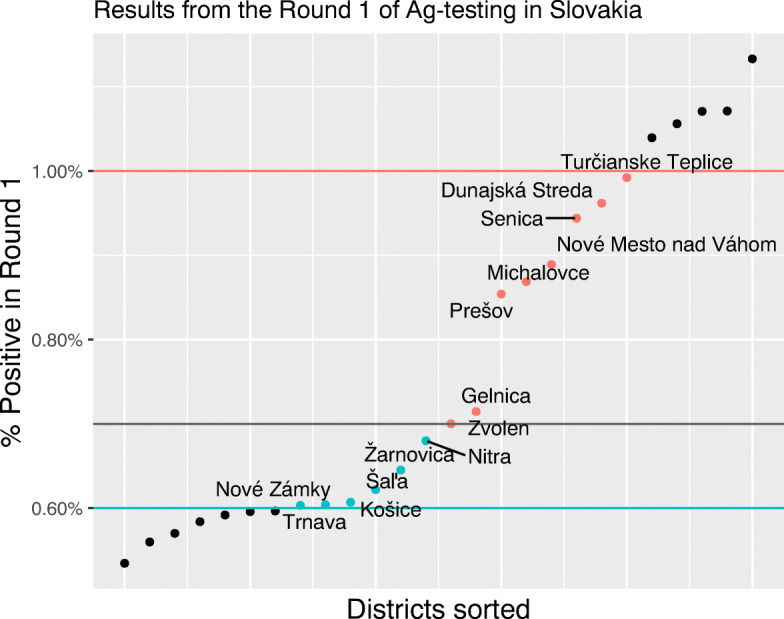
Distribution of districts by Round 1 test positivity in a restricted sample

As there is a high degree of arbitrariness in defining a “close” distance to the threshold, we restrict the sample to districts with comparable populations in treated and non-treated districts considering a range of cutoff points closer and farther from the threshold level of 0.7%. We first looked at a restricted group of treated districts where the share of positive tests in Round 1 was between 0.7 and 1%, representing the total population size of around 650,000. For our control group, we selected all districts where the share of positive tests lies in the range between 0.6 and 0.7%, representing the total population of approximately 750,000. The distribution of districts in our sample by test positivity in Round 1 and cutoff points 0.6% and 1% are presented in Fig. 4.

**Fig. 5:**
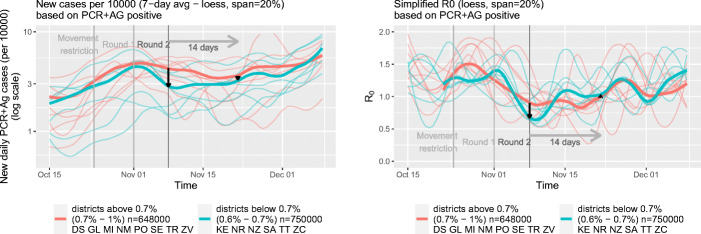
Evolution of infections and *R*_0_ in the districts below and above the threshold 0.7% for the restricted sample

The results for this restricted sample are presented in Table 3 and visualized in Fig. 5. We find that the effects of the second round of testing on the number of cases and *R*_0_ of a very similar magnitude as for the full sample. We estimate a reduction in new cases by around 35% which is very close to the 36% found in the full sample. When using *R*_0_ as an outcome, our the estimated impact (39%) is even slightly larger than in our full sample (see column 4). Given the reduction in the sample size, the standard errors increase and the estimated effects are estimated much less precisely, however.

**Table 3:** Effect of repeated mass testing on COVID-19 infections — restricted sample

	Dependent variable			
	Cases	log Cases	*R*_0_	log *R*_0_
	(1)	(2)	(3)	(4)
Tested in R2 × Post period	− 1.007	− 0.350	− 0.332^∗^	− 0.385^∗^
	(0.904)	(0.293)	(0.173)	(0.189)
Tested in R2	1.451^∗∗^	0.463^∗∗^	0.285^∗∗^	0.315^∗∗^
	(0.639)	(0.208)	(0.123)	(0.133)
Post period	0.328	0.136	0.328^∗∗^	0.395^∗∗∗^
	(0.616)	(0.200)	(0.118)	(0.128)
Intercept	2.769^∗∗∗^	0.941^∗∗∗^	0.699^∗∗∗^	− 0.378^∗∗∗^
	(0.435)	(0.141)	(0.083)	(0.091)
Observations	28	28	28	28
R^2^	0.193	0.181	0.286	0.318

Districts weighted by their population size

^∗∗∗^*p* < 0.01; ^∗∗^*p* < 0.05; ^∗^*p* < 0.1

A related concern in our setting might be the so-called *regression to the mean*, which refers to the situation when a unit’s repeated measurements are subject to a random error and our estimates might simply reflect that some districts with relatively high (low) disease prevalence experienced an “unlucky” (“lucky”) draw at the time of the second mass testing event and test positivity would have decreased or increased (reverted to the mean) regardless if the district was tested repeatedly or not (Barnett et al. 2005). In order to explore the sensitivity of our results to this possibility, we estimate a series of regressions for different sizes of the treatment and control groups. If regression to the mean was indeed a serious concern, then we would expect that our estimates vary significantly with the size of the populations included in our sample. In contrast, comparable effects regardless of the chosen sample size would suggest that regression to the mean effect is likely not a serious concern in our analysis.

**Fig. 6:**
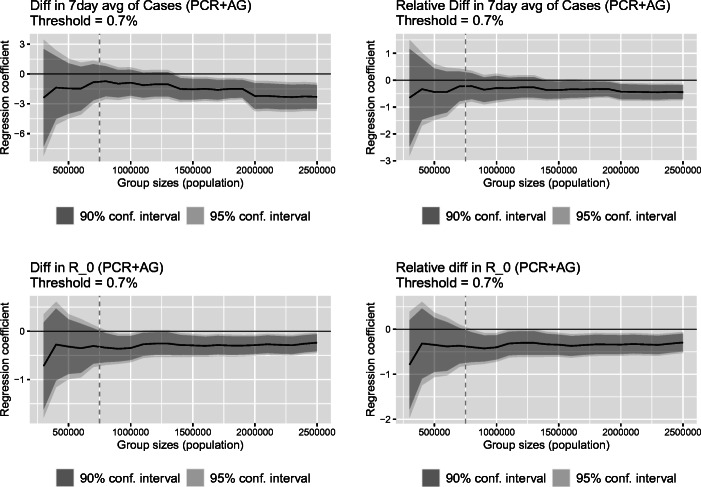
Estimated regression coefficient $\hat \beta _{1}$ with confidence intervals based on Eq. (1) as a function of the size of the groups below and above the threshold

Figure 6 presents the results. On the horizontal axis we plot the maximal size of both the treated and control group considered in the analysis, ranging from a sample with only a few included districts to the full sample. The black line denotes the respective regression coefficients of interest from Eq. (1) and the gray area depicts the 90% (darker) and 95% (brighter) confidence intervals.^23^ The gray vertical dashed line in the figure represent the results for the size of a cumulative population of 750,000, which is close to the choice made in our restricted sample presented in the previous section.^24^

From Fig. 6, one can see that our estimates for the impact of repeated mass testing on 7-day average of cases and its logarithm are rather stable across the varied sample sizes (top panels). We come to a similar conclusion when considering the sensitivity of our estimation results using *R*_0_ and its logarithm as the relevant outcome (bottom panels). Overall, given these results, we argue that regression to the mean is likely not of a major concern in our analysis.

Another noteworthy result shown in Fig. 6 is that after some thresholds of sample size, that is, as soon as a sufficient number of districts is included in the analysis, reducing the standard errors of the estimated effects, the estimated effects become statistically significant. These threshold are at about 1.4 million people included in the analysis of the 7-day average of cases and 0.8–0.9 million people included in the analysis of *R*_0_.

### Dynamic effects of repeated mass testing

Whether repeated mass antigen testing had a long-lasting impact or only short-lived effects on infections and the spread of the disease is relevant with respect to the assumption we made above about the lag of the measured effects after Round 2 (14 days) as well as its policy implications. Our initial evidence pointed toward a convergence in infections between our treatment and control group some time after the second round. In this section, we employ a more formal approach to look at the impact of repeated mass testing up to three weeks after retesting.

**Fig. 7:**
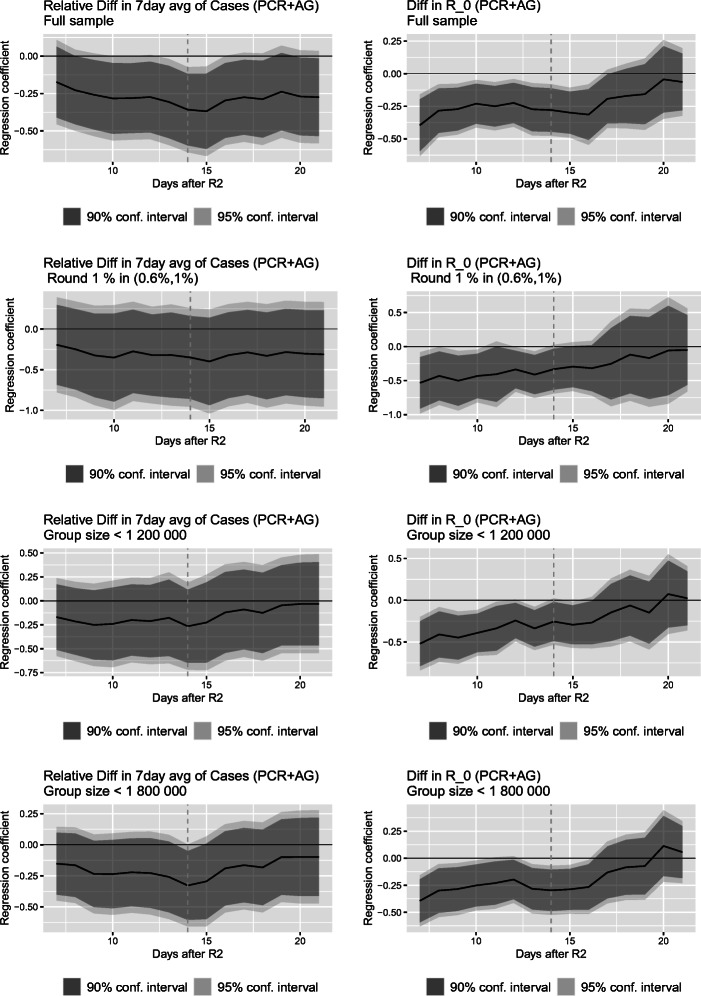
Estimated regression coefficient $\hat \beta _{1}$ with confidence intervals based on Eq. (1) as a function of time at which the outcome was measured. Dashed vertical line denotes two weeks after the re-testing round

For the sake of exposition, we depict our estimates graphically. Figure 7 visualizes the regression coefficients of interest for the 7-day average of cases and *R*_0_ as a function of the number of days after the second round of mass testing for four different specifications:

1. The full sample;
2. The restricted sample, that is, districts with a share of positive tests in the first round of mass testing between (0.6%, 1%) as described in Section 5.3;
3. Districts with a total population of less than 1,200,000 in our treatment and control group, respectively; and
4. Districts with a total population of less than 1,800,000 in our treatment and control group, respectively.

Considering the dynamic effects of repeated mass testing when using the full sample, presented in the first row of Fig. 7, two interesting features emerge. First, as can be seen from the top-left panel, repeated mass testing led to a fall in the share of positive cases, the effect peaking 15 days after the event. It also slowed down the spread of the disease as measured by *R*_0_ for a bit over two weeks after Round 2 (top right panel).

Second, after reaching the maximum reduction in cases 15 days after Round 2, the effect diminished toward the zero effect. This can be seen particularly strongly for the estimated effect on *R*_0_ presented in the top-right panel. About three weeks after the second mass testing, the estimated impact of repeated mass testing on *R*_0_ is statistically indistinct from zero.

We come to a similar conclusion when restricting our sample to districts closer to the threshold, presented in the second row of Fig. 7, or when making our treatment and control group comparable in terms of their populations, presented in rows three and four for total district populations above and below the threshold restricted to up to 1,200,000 and 1,800,000 citizens, respectively. In all these specifications, we estimate the maximum reduction in the numbers of cases roughly 15 days after the second mass testing event and a gradual reversal toward zero afterward. We find a similar but slightly stronger pattern for the impact of repeated mass testing on *R*_0_. Not surprisingly, the estimated confidence intervals are wider for these restricted samples; however, the estimated impacts retain statistical significance at 5% two weeks after Round 2 for *R*_0_ as well as the 7-day average of cases for samples with at least 1,800,000 citizens in each treated and non-treated districts. The results presented in this section imply that mass testing conducted only irregularly and after long time intervals is unlikely to be sustainably suppressing the pandemic.

## Robustness and falsification tests

To further test the salience of our results, we conducted a wide-range of additional robustness and falsification tests. For brevity’s sake, we only discuss the results here briefly and provide more details in Appendix 2.

First, we investigated the robustness of our results when relatively large districts, whose urban character might make them systematically differ from the rest of the sample, are removed form analysis. Urban districts also carry quite a lot of weight in our analysis and might therefore be the main driver of our results. Removing the most urbanized districts from the sample leads to very similar, and in the case of 7-day average infections, even stronger results than reported above. Using a different approach to this potential issue, we re-estimated the model without weights; this also lead to a similar conclusion.

Another concern might be that behavioral responses in the control or treated districts affect our results. Individuals living in districts subjected to only one mass testing event, for example, might behave less cautiously or be more mobile. Such behavioral adjustments might then confound our estimation results. To gauge this possibility we included the 7-day rolling average of Google workplace mobility as an additional control variable in our regression model.^25^ Even after including this indicator, results remain very similar to those presented in Section 5.

In our main analysis, we use the results from both PCR and antigen tests to minimize the measurement problems possibly caused by people substituting one type of testing for the other one. As discussed above, antigen testing might be more prone to delivering false results, however. To verify whether our results are impacted by this potential measurement error, we also consider test results from the more accurate PCR tests only. Using only PCR tests when calculating our outcome measures leads to very similar results as presented above.

We also looked at alternative and smoother measures of *R*_0_ in order to account for possible heterogenous intra-week testing dynamics in the different districts. The use of smoother measures led to a slight reduction of the estimated *R*_0_ coefficients from 0.3 to approximately 0.2.

Finally we conducted several placebo tests on our empirical analysis. First, we replace the true threshold of 0.7% with arbitrary false values of 1.2% and 0.5%. Second, we also vary the date of the second mass testing event, considering a false date one week earlier (November 1). As it turns out, we do not find any significant effects when considering these placebo specifications. These results provide further support to the credibility of our main findings.

## Conclusion

Repeated mass testing has been widely discussed by policy makers as a possible instrument that can be deployed to mitigate the spread of COVID-19 while also allowing the economy to remain open. Despite the attention, there is to date little empirical evidence if and how repeated mass testing might help.

We examine the effect of repeated mass testings on the spread of COVID-19 using a unique setting of mass antigen testing in Slovakia. Slovakia was the first country in the world conducting nationwide rapid antigen testing in autumn 2020. One day after the first mass testing event, the government announced that districts with an ex ante unknown and largely arbitrarily chosen share of positive tests of 0.7% or above had to undergo a second round of mass testing one week after the first round.

Exploiting this quasi-experimental setting in a difference-in-differences framework, our results suggest that 14 days after the second round of testing, new infections decreased by up to about 30% and *R*_0_ decreased by about 0.3. Investigating the patterns of these effects over time, we find that the impact on new infections peaked approximately two weeks after Round 2 and gradually faded out thereafter, with no significant impacts detectable three weeks after retesting.

While we think that our study makes a valuable contribution to the discussion about the potential benefits of mass testing, we also want to highlight its limitations. First, it is important to note that our estimated effects should be seen as the total effect of mass testing together with strict quarantine of all those who tested positive, their close or recent contacts, and those who chose not to get re-tested.

Second, our data does not allow us to distinguish other important channels possibly confounding our findings. For example, it its possible that individuals living in districts that participated in Round 1 of the testing gained only a false sense of security based on their good Round 1 results and, as a result, adhered less to social distancing measures or increased their risky behaviors. On the other hand, the signal of being selected for retesting might have increased the fear of infection in the respective districts, decreasing social contact and risky behaviors in those districts. The effects we estimate include the impacts of any such behavioral adjustments in treated and non-treated districts.

Third, from a public policy perspective, we do not evaluate the cost-effectiveness of mass testing; we do not look at its direct pecuniary and non-pecuniary costs, impacts on trust, the health care sector, or political risks surrounding intervention of such a large scale. Whether and under what conditions a mass testing strategy is feasible, cost-effective, or whether it would be the best alternative for suppressing the pandemic are important questions that we do not address in this study and that must be considered before implementing any mass testing strategy.

Overall, we see our study as an early contribution to our understanding of whether and how mass testing can contribute to the suppression of pandemics. While the emerging availability of an effective vaccination could be seen as undermining the usefulness of mass testing presently, new variants of the virus could render currently available vaccinations ineffective and entirely new diseases may start new pandemics. Our study hence offers useful lessons from the current pandemic about the possible role of mass testing also for future occurrences. Addressing the remaining questions mentioned above is a fruitful area for future research.

## Figures and Tables

**Fig. 8:**
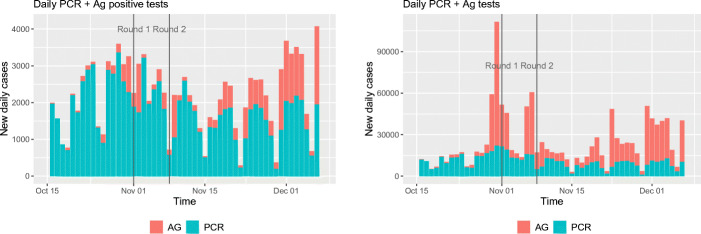
Evolution of positive tests and the number of administered tests

**Fig. 9:**
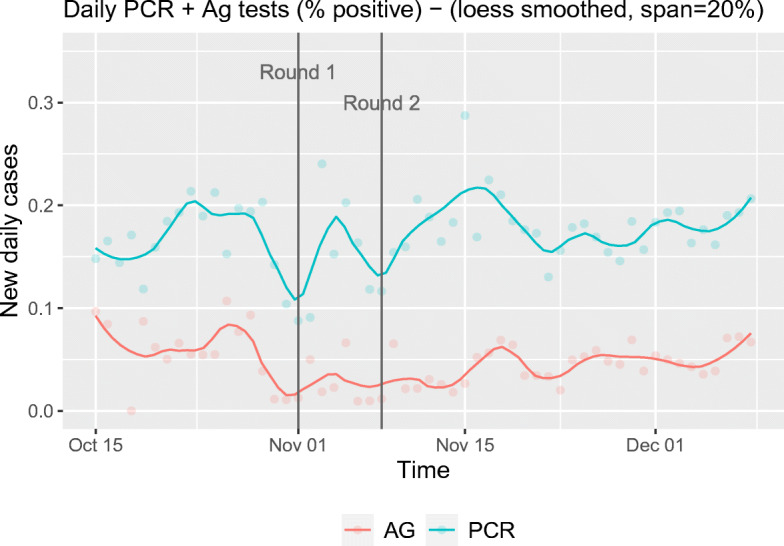
Evolution of percent positive for PCR and Ag tests

**Fig. 10:**
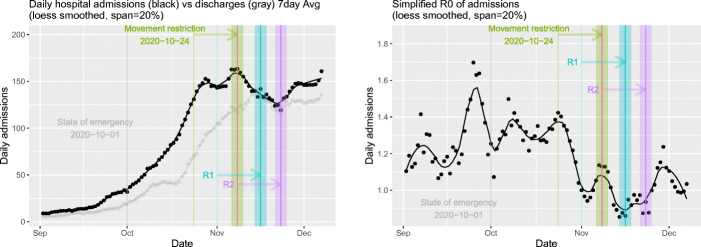
Hospital admissions, discharges and *R*_0_. Arrows point 15 days ahead

**Fig. 11:**
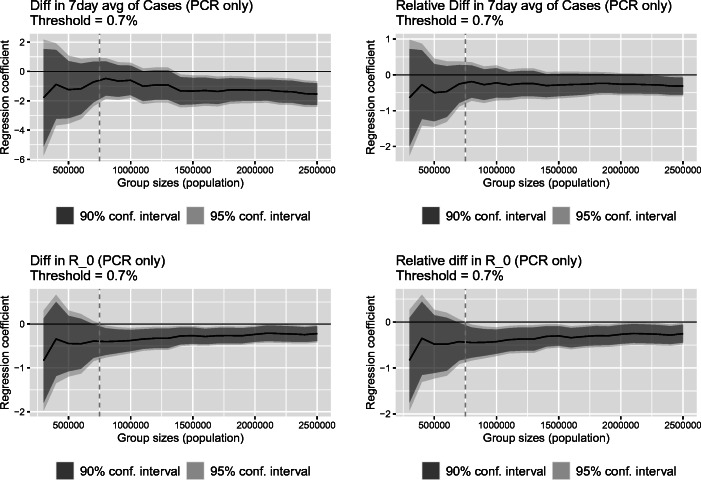
Estimated regression coefficient $\hat \beta _{1}$ with confidence intervals based on Eq. (1) as a function of the size of the groups below and above the threshold. PCR tests only were used in calculations

**Fig. 12:**
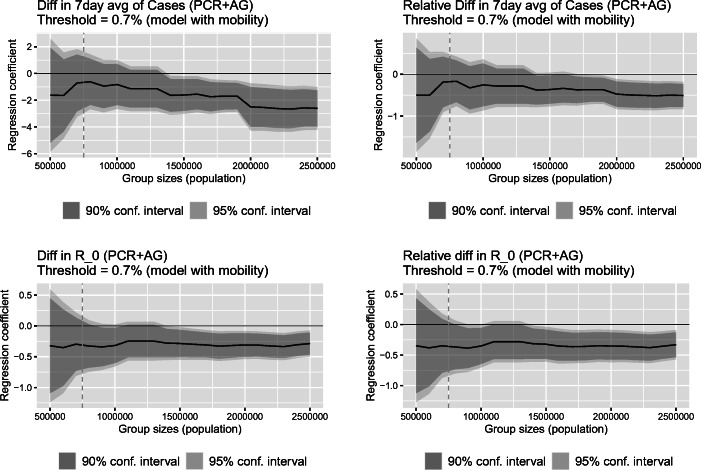
Estimated regression coefficient $\hat \beta _{1}$ with confidence intervals based on Eq. (2) as a function of the size of the groups below and above the threshold

**Fig. 13:**
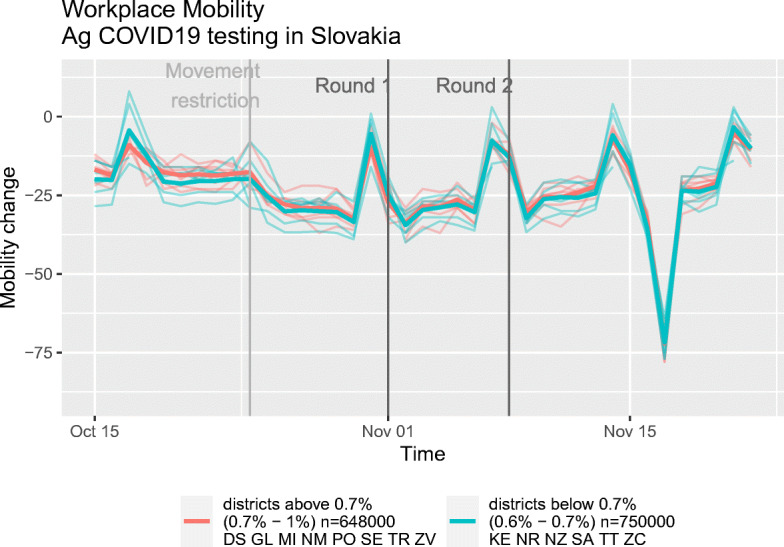
Google mobility data — Workplace

**Fig. 14:**
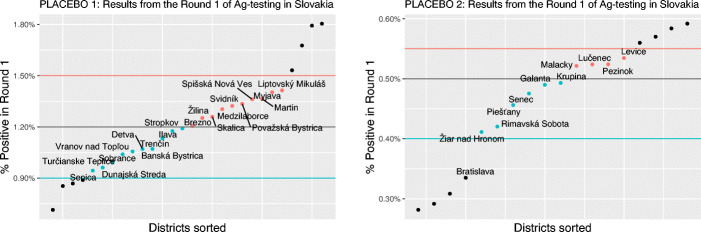
Choice of districts in the placebos

**Fig. 15:**
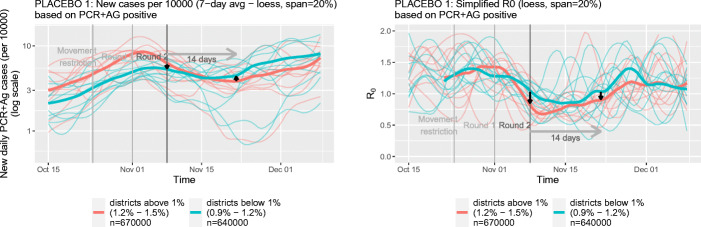
Evolution of infections and *R*_0_ in the districts below and above the Placebo threshold 1.2%

**Fig. 16:**
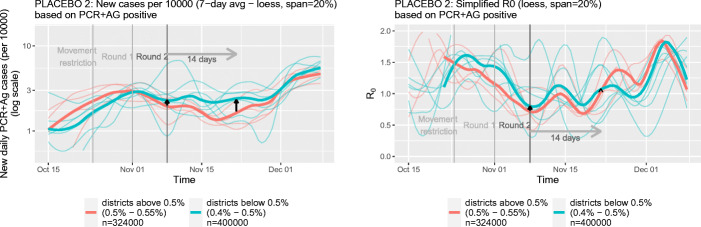
Evolution of infections and *R*_0_ in the districts below and above the Placebo threshold 0.5%

**Fig. 17:**
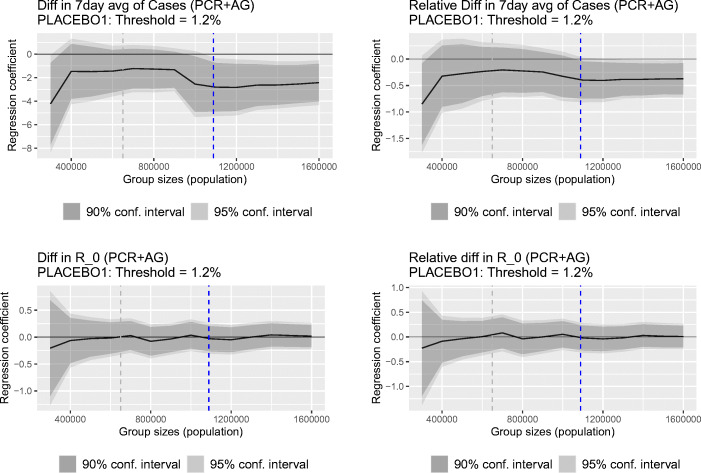
Regression coefficient as a function of maximal size of the groups below and above the threshold. Vertical dash line stands for the size of the placebo specification groups and vertical blue line depicts the size of the below group that does not include any district that was not tested in Round 2. The results to the left of this blue line are all tested in Round 2

**Fig. 18:**
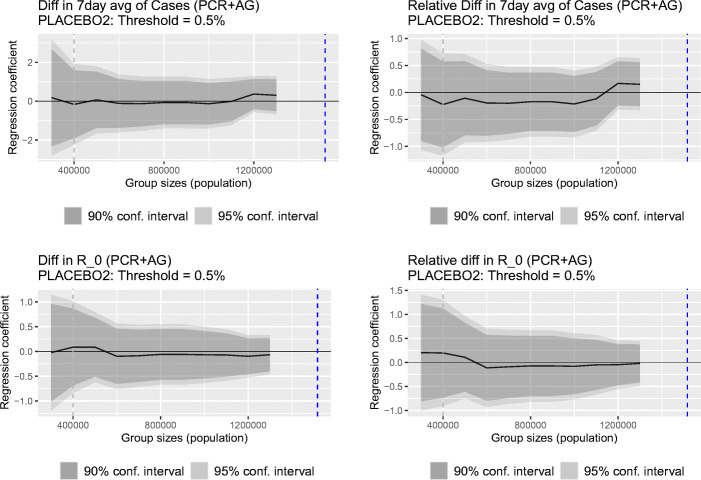
Regression coefficient as a function of maximal size of the groups below and above the threshold. The vertical dash line stands for the size of the placebo specification groups and the vertical blue line depicts the size of the above group that does not include any district that was tested in Round 2. The results to the left of this blue line are all based on districts not tested in Round 2

**Fig. 19:**
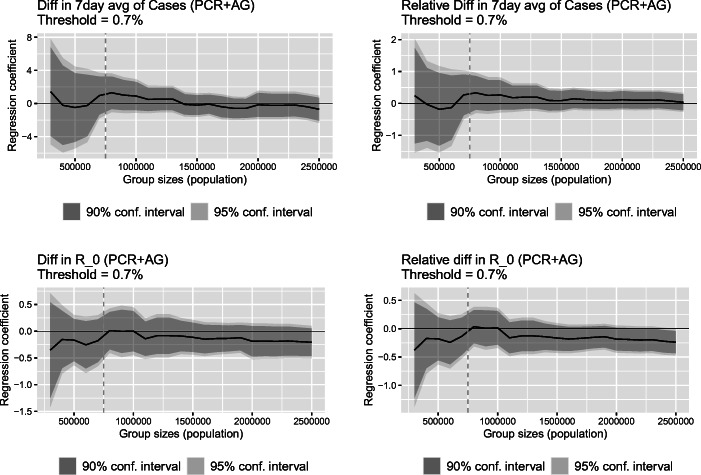
Estimated regression coefficient $\hat \beta _{1}$ with confidence intervals based on Eq. (1) as a function of the size of the groups below and above the threshold. Round 2 date was (incorrectly) set for Nov 1

## Appendix: 1. Additional information on PCR and antigen testing

In this section, we give a brief overview over the prevalence of PCR and antigen tests over time. We also provide an overview over the number of positive tests as well as numbers of hospitalizations over time.

As it is apparent from Fig. 8, the number of positive PCR tests went down after the mass testing. At the same time, we see an increase in positive antigen tests. Around mid October, antigen testing sites were introduced at various places in Slovakia, where it was possible to get tested for free. The availability of these free antigen tests increased over time as demonstrated in the right pane of Fig. 8. With the wider availability, it is likely that a larger share of the population has switched to antigen tests, rather than the more expensive PCR tests. Given that there was a large variation in the antigen testing capacities on the district level, we included antigen tests into our analysis.

Figure 9 shows that the positivity of PCR tests went up in the week after the Round 2 of testing and the went down the week after. The positivity of the antigen tests appeared to be somewhat more stable with a spike approximately 10 days after the second round of testing.

Hospitalizations (Fig. 10) decreased around the weekend of the second round of antigen testing. The right pane shows the simplified *R*_0_ of hospital admissions. From these figures alone, it is not possible to disentangle the potential effect of the antigen testing as several other policy measures were in place, such as school closures and movement restrictions. However, we see some improvement in the *R*_0_, which fell below 1 for approximately two weeks. Data on hospitalizations are independent of the testing capacities and therefore contain a lot of information about the epidemic situation although with a time lag.^26^

## Appendix: 2. Additional robustness and falsification tests

This section presents details on all the sensitivity and robustness checks described in Section 6. For convenience, we provide the summary of all the results from our robustness checks in Tables 4 (full sample) and 5 (restricted sample).

**Table 4:** Full sample: Sensitivity overview

	Dependent variable			
	Cases	log Cases	R0	log R0
	(1)	(2)	(3)	(4)
Original estimates	− 2.252^∗∗∗^	− 0.358^∗∗^	− 0.279^∗∗∗^	− 0.314^∗∗∗^
	(0.632)	(0.144)	(0.100)	(0.112)
w/o Bratislava district	− 2.376^∗∗∗^	− 0.423^∗∗∗^	− 0.214^∗∗^	− 0.265^∗∗^
	(0.683)	(0.154)	(0.106)	(0.118)
w/o Košice district	− 2.279^∗∗∗^	− 0.365^∗∗^	− 0.271^∗∗∗^	− 0.301^∗∗∗^
	(0.652)	(0.148)	(0.103)	(0.115)
w/o Bratislava and Košice districts	− 2.428^∗∗∗^	− 0.442^∗∗∗^	− 0.195^∗^	− 0.301^∗∗∗^
	(0.713)	(0.160)	(0.111)	(0.115)
Unweighted	− 1.790^∗∗^	− 0.312^∗^	− 0.133	− 0.194
	(0.694)	(0.173)	(0.125)	(0.138)
PCR only	− 1.654^∗∗∗^	− 0.249^∗^	− 0.247^∗∗∗^	− 0.266^∗∗^
	(0.450)	(0.138)	(0.093)	(0.111)
Considering mobility	− 2.537^∗∗∗^	− 0.415^∗∗∗^	− 0.310^∗∗∗^	− 0.329^∗∗∗^
	(0.719)	(0.154)	(0.108)	(0.121)
R0 based on 7 day avg			− 0.213^∗∗∗^	− 0.216^∗∗∗^
			(0.078)	(0.078)
7 day avg of R0			− 0.204^∗∗^	− 0.196^∗∗^
			(0.085)	(0.079)
Date of Second Mass Testing (Nov 1)	− 0.749	0.120	− 0.041	− 0.101
	(0.703)	(0.142)	(0.144)	(0.115)

This table presents the results for the key parameter *β*_1_ from Eq. (1) estimated on a full sample with population sizes used as weights. Rows 2 to 5 show the results when excluding large urban districts from our analysis and using unweighted regressions respectively (Appendix A2.1). Row 6 presents the results considering only test results from PCR data only (Appendix A2.2) and row 7 shows the results when including workplace mobility in the estimation (Appendix A2.3). Rows 8 and 9 present results based on R0 measure calculated on a 7-day average of infection cases and on a 7-day average of R0 respectively (Appendix A2.4). Finally, the last row shows estimates from placebo regression using a different date (Appendix A2.5)

Districts weighted by their population size

^∗∗∗^*p* < 0.01; ^∗∗^*p* < 0.05; ^∗^*p* < 0.1

**Table 5:** Restricted sample: Sensitivity overview

	Dependent variable			
	Cases	log Cases	R0	log R0
	(1)	(2)	(3)	(4)
Original model	− 1.007	− 0.350	− 0.332^∗^	− 0.385^∗^
	(0.904)	(0.293)	(0.173)	(0.189)
w/o Košice district	− 1.347	− 0.492	− 0.320	− 0.361
	(0.996)	(0.324)	(0.198)	(0.215)
Unweighted	− 1.427	− 0.519	− 0.340	− 0.411
	(0.968)	(0.336)	(0.222)	(0.257)
PCR only	− 0.741	− 0.301	− 0.387^∗∗^	− 0.420^∗∗^
	(0.706)	(0.277)	(0.179)	(0.191)
Considering mobility	− 0.926	− 0.319	− 0.319	− 0.360^∗^
	(1.021)	(0.320)	(0.193)	(0.201)
R0 based on 7 day avg			− 0.191	− 0.171
			(0.160)	(0.148)
7 day avg of R0			− 0.152	− 0.138
			(0.175)	(0.158)
Placebo regression — Threshold 1.2%	− 1.325	− 0.229	− 0.052	− 0.005
	(0.989)	(0.246)	(0.160)	(0.186)
Placebo regression — Threshold 0.5%	− 0.298	− 0.242	0.057	0.121
	(0.795)	(0.372)	(0.370)	(0.436)
Date of second mass testing (Nov 1)	0.470	0.145	− 0.109	− 0.082
	(1.141)	(0.312)	(0.258)	(0.201)

This table presents the results for the key parameter *β*_1_ from Eq. (1) estimated on restricted samples (visualized on Fig. 4) with population sizes used as weights. Row 2 and 3 show the results when excluding large urban districts from our analysis and using unweighted regressions respectively (Appendix A2.1). Row 4 presents the results considering only test results from PCR data only (Appendix A2.2) and row 5 shows the results when including workplace mobility in the estimation (Appendix A2.3). Rows 6 and 7 present results based on R0 measure calculated on a 7-day average of infection cases and on a 7-day average of R0 respectively (Appendix A2.4). Finally, the last three rows show estimates from placebo regressions using arbitrary thresholds (using samples depicted in Fig. 14) or dates (Appendix A2.5)

Districts weighted by their population size

^∗∗∗^*p* < 0.01; ^∗∗^*p* < 0.05; ^∗^*p* < 0.1

### A2.1 Sensitivity to district population

A concern in our analysis is that districts subjected to one or two rounds of mass testing substantially differ in terms of population density size. We assess the sensitivity of our results by first removing the most urban districts with the highest population density (Bratislava and Košice). Doing so yields somewhat stronger results for infections (levels and logs) and slightly weaker effects on *R*_0_ in both the full and restricted sample. In the restricted sample, removing the Košice district increased the size of the coefficient for logarithm of cases.^27^

We also tested the sensitivity of our estimates to district population size by not weighting the regressions. We obtain qualitatively very similar although slightly smaller effects compared to our baseline estimates considering the full sample. In the restricted sample, we even obtain slightly stronger effects, although they are not statistically significant at the conventional level. Nevertheless, the results from this exercise supports the conclusion made in the main part of our paper and our expectation that reducing the sample size will increase standard errors of our estimates. Given the large differences between district sizes, we believe that the results from an analysis with population weights are more representative of the true effect.

### A2.2 Results based on PCR tests only

As discussed in the main part of our paper, PCR and antigen tests can differ in terms their sensitivity and specificity as well as with logistics associated with their administration. While PCR testing imposes a larger administrative burden, such as a time delay when receiving the results, they tend to suffer less from false tests results. Therefore, when pooling results from PCR and antigen tests together, our outcome variables might be subjected to different types of measurement error.

In row 7 labelled “PCR only” of Tables 4 and 5, we present results when only considering PCR tests in our estimation. We report the results for different sizes of the reference groups in Fig. 11. In general, the estimated effect falls slightly when compared to our original estimates. As the testing campaign was increasingly build on rapid antigen tests and positive test results were not confirmed using PCR tests, these results might underestimate the benefits of repeated mass testing. In general, even when only considering PCR test results, our estimates still point toward the short-term benefits of repeated mass testing.

### A2.3 Model accounting for workplace mobility

Behavioral response to being subject to one or two mass testing events might be another important channel which can at least partly explain our results. Individuals living in districts with only one mass testing event might as a result become less careful when interacting with others, as the low test positivity in Round 1 might have led to a false sense of security. We indirectly explore the importance of behavioral channels by including mobility patterns on the district level as an additional variable in our model. We mainly concentrate on *Workplace* mobility, as the rate of missing data on the district level was about 50% for other types of Google mobility measures, such as (*Retail and Recreation*, *Grocery and Pharmacy*, *Parks*, *Transit Stations*, *Residential*).

Including workplace mobility, our model results in the following specification:

2 $$ y_{it} = \beta_{0} + \beta_{1} (testedR2_{i} \cdot t) + \beta_{2} t + \beta_{3} testedR2_{i} + \beta_{4} mob_{it}+ \epsilon_{it}, $$

where *m**o**b*_*i**t*_ stands for the 7-day rolling average of Google workplace mobility measure at *t* = 0 (Nov 8, 2020) and *t* = 1 (Nov 22, 2020) (Google LLC 2021).

Including mobility patterns in our model affects our estimates only slightly (see Fig. 12). Considering the full sample, the estimated effects are even somewhat stronger. The limited impact of the inclusion of mobility on our estimates is not surprising given the little differences in the mobility patterns across districts (see Fig. 13).

### A2.4 Sensitivity to alternative measures of *R*_0_

The simplified measure of *R*_0_ that we use in our main analysis is based on the raw daily cases. Such a measure may be highly variable, especially in smaller districts. It might also be affected by different testing schedules during the weekend. Given these possible concerns, we also consider two alternative specifications of *R*_0_ which likely provide more stable and smoother measures of epidemic growth. The first one is *R*_0_ calculated from the 7-day average of infections. The second measure is the 7-day average of *R*_0_ calculated from the raw cases. Notice, however, that such smoothing is expected to attenuate the effect. This attenuation is also reflected in our estimates. The cost of having a smoother measure of *R*_0_ comes at the price of a reduced variability in the outcome variable. In the full sample, the estimated coefficients drop from approximately 0.3 to 0.2 and the decrease is even more pronounced in the restricted sample (see Tables 4 and 5). Nevertheless, our results with these new measures still point toward some short-term benefits of mass testing.

### A2.5 Placebo tests for threshold and date

For the last robustness exercises, we evaluate the effects for “placebo” testing thresholds and a “placebo” testing date. We first consider placebo thresholds above and below the original one of 0.7%. We present the results for 1.2% and 0.5% placebo thresholds. These two false thresholds are then used to split the sample into false treatment or control groups. Around each of these two new thresholds we define our new sample, similar as in Section 5.3. The choice of the districts are depicted in Fig. 14 for the new higher threshold of 1.2% consisting of only districts that were tested in Round 2 (left-hand side) and for the new lower threshold of 0.5% consisting of only districts that were not tested in Round 2 (right-hand side). Given that in these placebo tests there is no variation in participation in the second round of testing, the placebo tests should yield no effects.

The results are presented in rows 8 and 9 of Table 5. Reassuringly, in our placebo regressions we do not find any effect, which supports our original results. This can also be seen from a simple comparison as depicted in Figs. 15 and 16. We also investigated the sensitivity of our results to the chosen group size (see also the discussion in Section 5). The estimates are shown in Figs. 17 and 18 for the higher and lower threshold respectively. It is reassuring, that the placebo tests generally show insignificant results; the only exception being the placebo test for the 7-day average of cases but only after the placebo test is contaminated by the inclusion of districts which were not tested in Round 2 (to the right of the vertical blue dashed line). As it turns out, we do not find evidence that our placebo results were driven by the chosen group size.

Lastly, we also run a regression with a placebo date of Round 2. We do so by setting the new date to Nov 1, one week prior to the actual Round 2 of mass testing. As before, we measure our outcome 14 days after this date, on Nov 15. Figure 19 shows that the estimated effects for this placebo test for different sizes of the reference groups. Our estimates are small and mostly not statistically different from zero at any conventional level. We interpret this as a sign that there was no anticipation of the effect of the actual Round 2, supporting our identifying assumptions.

Overall, we see the results from the robustness checks in this section as an indirect evidence that results from our original model with the threshold 0.7% and date of Round 2 as Nov 8 are not due to some lucky draw, but rather to an effect of the second round of mass testing.

## Footnotes

1 The data was taken from https://coronavirus.jhu.edu/.

2 Whereas a mass COVID-19 vaccination strategy to re-open the economy will hopefully be available in the future, delivery shortages, the possibility of virus mutations (or the emergence of entirely new viruses) reducing the efficacy of existing vaccines, and uncertainty about the share of the public that is willing to get vaccinated justify an evaluation of alternative strategies, such as mass testing.

3 As we discuss in more detail in Section 4, due to the small sample size, employing a non- or semi-parametric regression discontinuity design is not feasible in our setting.

4 Fortunately, the use of a single type of rapid antigen test (SD-Biosensor Standard Q) in the mass testing campaign mitigates the risk of systematic differences in sensitivity and specificity of testing across districts. Given the relatively large numbers of tests administered daily and the time-invariant regulation of passive surveillance testing protocols during the period studied, we consider the risk of systematically changing differences between populations tested in districts above and below the threshold relatively low.

5 There are many studies examining the impact of the COVID-19 pandemic on different types of outcomes, such as inequality in financial well-being (Botha et al. 2021), the effect on the US presidential election (Baccini et al. 2021), and labor demand (Kahn et al. 2020); see also, for example, Brodeur et al. (2020) for a review.

6 Similar ideas are explored in a blog post by Šuster (2021), who compares COVID-19 trends in the districts that were tested and those that were not tested in the second round of mass antigen testing in Slovakia.

7 State-sponsored surveillance antigen testing, mass testing, and prescribed PCR testing (for symptomatic people, people returning from abroad, etc.) were provided free of charge.

8 See RTVS (2020) and Pravda (2020)

9 See SME (2020) and DenníkN (2020a)

10 DenníkN (2020b)

11 Our results remain virtually unchanged when including the four pilot districts into our analysis.

12 The data is available under https://github.com/Institut-Zdravotnych-Analyz/covid19-data

13 The IHA merges PCR test results for 9 districts in the two largest cities — Bratislava I, II, III, IV, V and Košice I, II, III, IV into one large district for Bratislava and Košice, respectively. We therefore also merged the Ag test results for these districts and work with 72 districts instead of 79.

14 The IHA also provides data on the number of hospital admissions. There are some problems with this information, however. For example, patients are admitted to the nearest hospital, which may or may not be located in the same district. In fact, there are some districts that do not have a hospital. Therefore, the numbers of district hospitalizations do not reflect the epidemiological situation on the district level accurately. We therefore decided not to use hospitalization data in our analysis.

15 We note that the measure of *R*_0_ is a simplified version and there are alternative ways how *R*_0_ can be estimated, (Abbott et al. 2020). We provide results using alternative measures of *R*_0_ in Appendix A2.4.

16 The participation rate is calculated relative to the overall population and not the eligible one, as discussed in the previous section. The data does not allow us to break down the participation rate by different characteristics.

17 The 7-day rolling average of new infection cases is defined as $\left ({\sum }_{\tau =T-6}^{T} y_{\tau } \right ) $ where *T* is the date — Nov 8, 2020 for *t* = 0 and Nov 22, 2020 for *t* = 1.

18 All data transformations, graphics, and statistical analyses in this study were conducted using R (R Core Team 2020).

19 The parameter *β*_2_ captures the average difference in outcomes between *t* = 1 and *t* = 0 for the non-tested districts and *β*_3_ stands for the average pre-treatment difference in outcomes between the tested and non-tested districts.

20 False positivity or false negativity of testing is permissible, however, as long as these errors do not systematically and differently vary within treated and non-treated districts over time.

21 We are grateful to a referee for pointing this out.

22 Using unweighted regressions leads to qualitatively similar results.

23 More precisely, we sequentially include districts in the control and treatment group until the cumulative population in these groups crosses a specified threshold.

24 These results are not completely the same, as the range of percentages that correspond to this line would not consist of round numbers, such as 0.6% and 1% in the restricted sample.

25 This measure quantifies the changes at which people with Android smartphones visit places that are labelled by Google as *workplace*. Other mobility trends reported by Google at the required level of granularity have many missing values and are therefore not suitable for our analysis. See Google LLC (2021) for more details.
